# Penile involvement associated with renal pelvic squamous cell carcinoma: a case report and mechanistic considerations

**DOI:** 10.3389/fonc.2026.1761194

**Published:** 2026-02-27

**Authors:** Kotaro Masaki, Takuya Tsujino, Hiroyuki Okada, Yuki Yoshikawa, Ryoichi Maenosono, Yusaku Imura, Masashi Sanada, Kensuke Hirosuna, Yuta Furusawa, Rei Yoshimi, Issei Kojima, Moritoshi Sakamoto, Kengo Iwatsuki, Yuki Nakajima, Takuya Matsuda, Takuya Higashio, Shuya Tsuchida, Shogo Yamazaki, Ko Nakamura, Tatsuo Fukushima, Kazuki Nishimura, Keita Nakamori, Takeshi Tsutsumi, Tomohisa Matsunaga, Haruhito Azuma

**Affiliations:** Department of Urology, Osaka Medical and Pharmaceutical University, Takatsuki, Japan

**Keywords:** case report, pelvic cancer, penile metastasis, retrograde venous spread, squamous cell carcinoma

## Abstract

**Background:**

Squamous cell carcinoma (SCC) of the renal pelvis is an uncommon malignancy, accounting for less than 1% of upper urinary tract tumors. Penile metastasis from renal pelvic SCC has not been documented.

**Case presentation:**

A 74-year-old man presented with a firm penile nodule. Magnetic resonance imaging (MRI) demonstrated an intracavernosal mass, while contrast-enhanced computed tomography (CT) revealed a large left renal pelvic tumor (89 mm) with hepatic and hilar lymph node metastases, without pelvic or inguinal lymphadenopathy. Histopathological examination of both the renal pelvic and penile lesions showed keratinizing SCC. Immunohistochemistry demonstrated diffuse p40 and p63 positivity with PAX8 and p16 negativity, supporting a urothelial tract origin rather than a primary penile carcinoma. Given the disseminated disease and rapid clinical deterioration, no systemic or surgical therapy was undertaken, and best supportive care was provided.

**Conclusion:**

This case constitutes, to our knowledge, the first reported instance of penile involvement most consistent with metastatic renal pelvic SCC. In patients with advanced upper urinary tract malignancy who develop penile lesions, secondary involvement should be considered. The absence of regional lymphadenopathy and the disseminated pattern suggest a hematogenous retrograde venous dissemination pathway.

## Introduction

Squamous cell carcinoma (SCC) of the renal pelvis is an uncommon histologic subtype of upper urinary tract cancer, accounting for approximately 0.5%–8% of renal pelvic malignancies, with reported incidence varying widely among studies and populations (1, 2). It typically arises in association with chronic irritation due to renal calculi, infection, or hydronephrosis, leading to squamous metaplasia and subsequent malignant transformation (1). Owing to its indolent onset and nonspecific symptoms, the disease is often diagnosed at an advanced stage and carries a poor prognosis. Penile metastasis is an exceptionally rare manifestation of disseminated malignancy and most frequently originates from genitourinary primaries such as prostate and bladder carcinoma (3). Penile involvement associated with renal pelvic SCC has not been previously described. Here, we report the first documented case of penile involvement most consistent with metastatic renal pelvic SCC and explore potential metastatic routes and pathological implications.

## Case presentation

**Figure 1:**
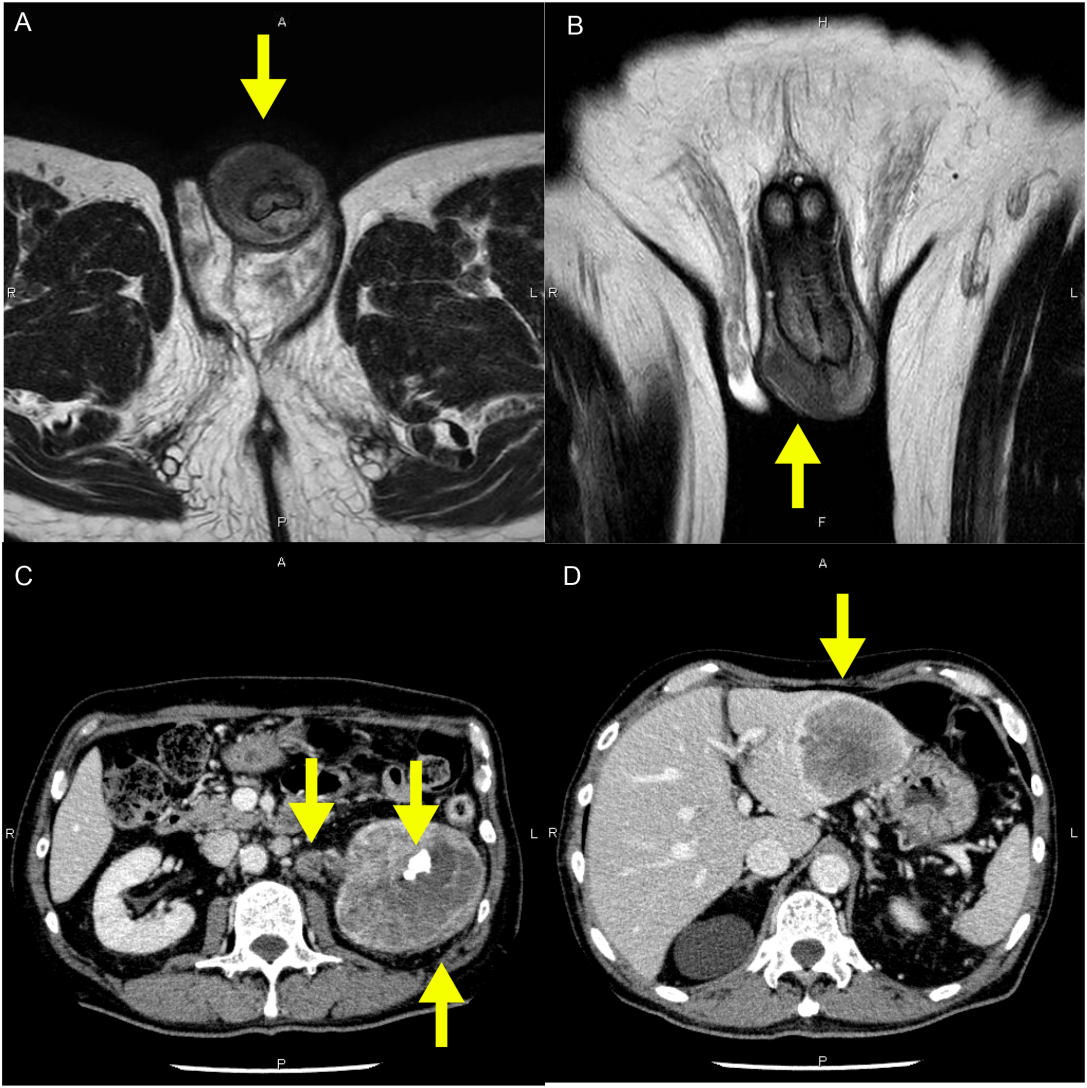
Magnetic resonance imaging (MRI) and contrast-enhanced computed tomography (CT) findings. **(A, B)** T2-weighted MRI of the penis in axial **(A)** and coronal **(B)** views showing a well-enhancing mass in the right glans (yellow arrows). **(C, D)** Contrast-enhanced abdominal CT demonstrating a large left renal pelvic mass with a renal calculus and hilar lymph node enlargement **(C)** and hepatic metastases **(D)**. Yellow arrows indicate tumors, calculus, and metastatic lesions.

**Figure 2:**
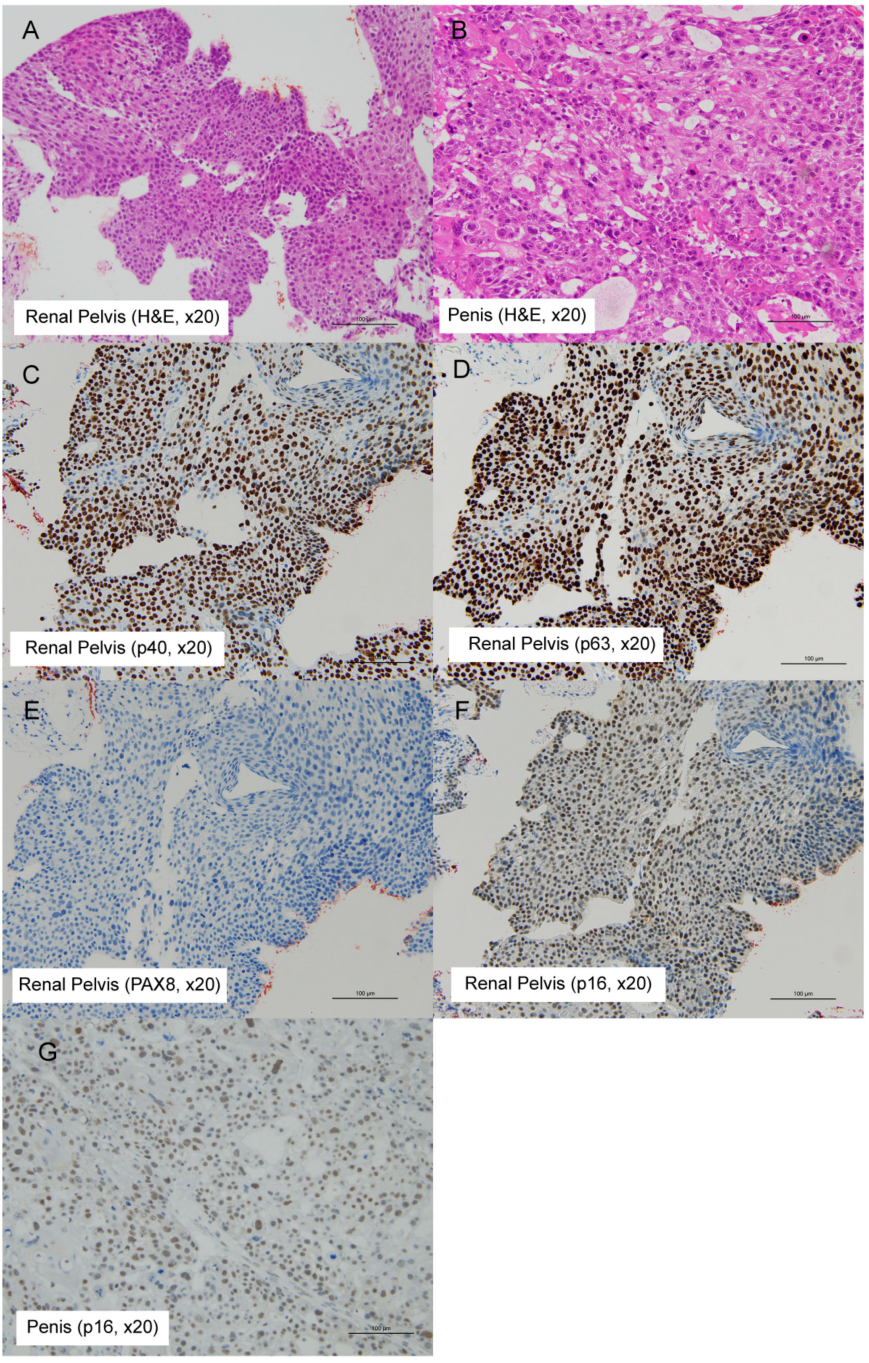
Histopathologic and immunohistochemical findings of the renal pelvic and penile lesions. **(A, B)** Hematoxylin and eosin (H&E) staining (×20) of the renal pelvic tumor **(A)** and the penile lesion **(B)**, both showing keratinizing squamous cell carcinoma. **(C, D)** Immunohistochemical staining (×20) of the renal pelvic tumor demonstrating strong diffuse nuclear positivity for p40 **(C)** and p63 **(D)**. **(E, F)** Immunohistochemical staining (×20) of the renal pelvic tumor showing absence of nuclear staining for PAX8 **(E)** and p16 **(F)**. **(G)** Immunohistochemical staining (×20) of the penile lesion showing absence of p16 expression. No nonspecific background staining was observed.

**Figure 3:**
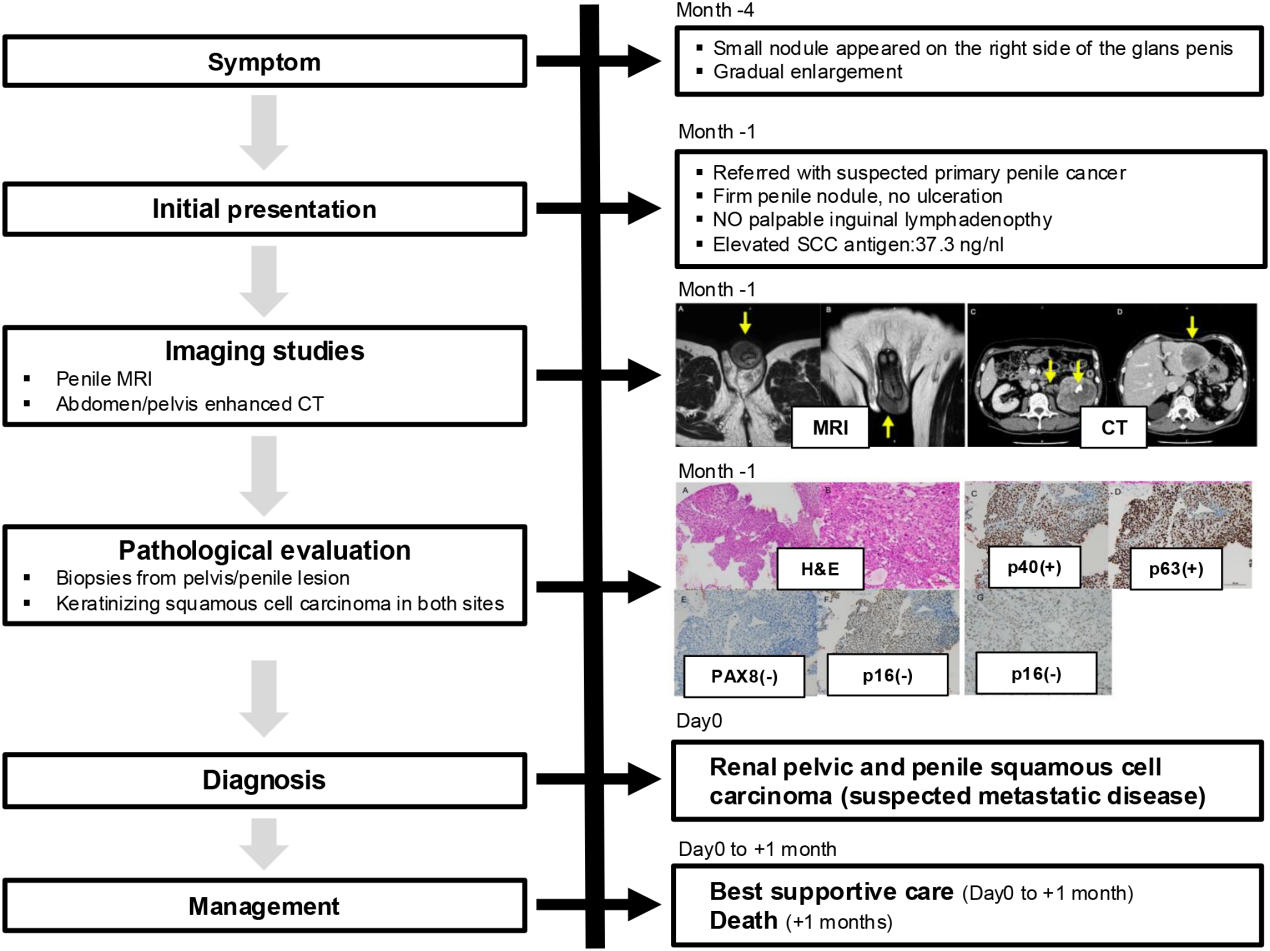
Timeline summarizing the clinical course of the patient. A penile nodule was first noted approximately 4 months before presentation (Month −4), followed by gradual enlargement. At 1 month before diagnosis (Month −1), the patient was referred to our institution, where penile MRI and contrast-enhanced abdominal CT were performed, revealing a penile mass and a large left renal pelvic tumor with distant metastases. Histopathological evaluation of biopsies from the renal pelvis and penile lesion demonstrated keratinizing squamous cell carcinoma. At diagnosis (Day 0), the condition was assessed as renal pelvic and penile squamous cell carcinoma, suspicious for metastatic disease. Given the advanced disease and rapid clinical deterioration, best supportive care was selected, and the patient died 1 month after diagnosis.

A 74-year-old man presented with a rapidly enlarging nodule on the right side of the glans penis that had developed over 4 months. He was referred to our institution with a presumptive diagnosis of primary penile carcinoma. Past medical history included hypothyroidism and appendectomy. Family history and psychosocial history were unremarkable or unavailable. On physical examination, a firm nodule was palpated in the right glans; no ulceration was evident, and no palpable inguinal lymphadenopathy was identified. Laboratory evaluation revealed an elevated SCC antigen (37.3 ng/mL). Magnetic resonance imaging (MRI) of the penis demonstrated a well-enhancing mass within the glans (**Figures 1A, B**). Contrast-enhanced computed tomography (CT) of the abdomen revealed a large left renal pelvic mass measuring 89 mm, with a renal stone, hepatic metastases, and hilar lymphadenopathy, without pelvic or inguinal lymphadenopathy (**Figures 1C, D**). Ureteroscopy confirmed a solid tumor occupying the left renal pelvis. A biopsy specimen was obtained from the renal pelvic lesion for histopathologic evaluation. A biopsy of the penile lesion was performed. Histopathological examination of both renal pelvic and penile lesions demonstrated keratinizing SCC (**Figures 2A, B**). Immunohistochemistry revealed p40 and p63 positivity, with PAX8 and p16 negativity (**Figures 2C–G**). Given the synchronous advanced renal pelvic SCC, concordant histology between both sites, and the absence of regional lymphadenopathy, the penile lesion was considered most consistent with metastatic involvement; however, a synchronous primary penile SCC cannot be completely excluded. A key diagnostic challenge was distinguishing metastatic involvement from a synchronous primary penile SCC. Because of the disseminated disease burden and rapid clinical decline, no surgical or systemic anticancer therapy was initiated, and best supportive care was provided. The patient’s condition rapidly declined, and he died 1 month after diagnosis. The overall clinical course is summarized in **Figure 3**.

## Pathological and molecular findings

Microscopic examination of both lesions revealed keratinizing SCC with intercellular bridges. Immunohistochemically, tumor cells expressed p40 and p63, confirming squamous differentiation (4), while PAX8 negativity excluded renal cell carcinoma (5). The lack of p16 expression indicated an HPV-independent pathogenesis (6); however, p16 is an imperfect surrogate marker, and HPV-independent primary penile SCC remains possible. Assessment of ureteral involvement was not feasible because histopathological evaluation was limited to biopsy specimens from the renal pelvic lesion.

## Discussion

**Figure 4:**
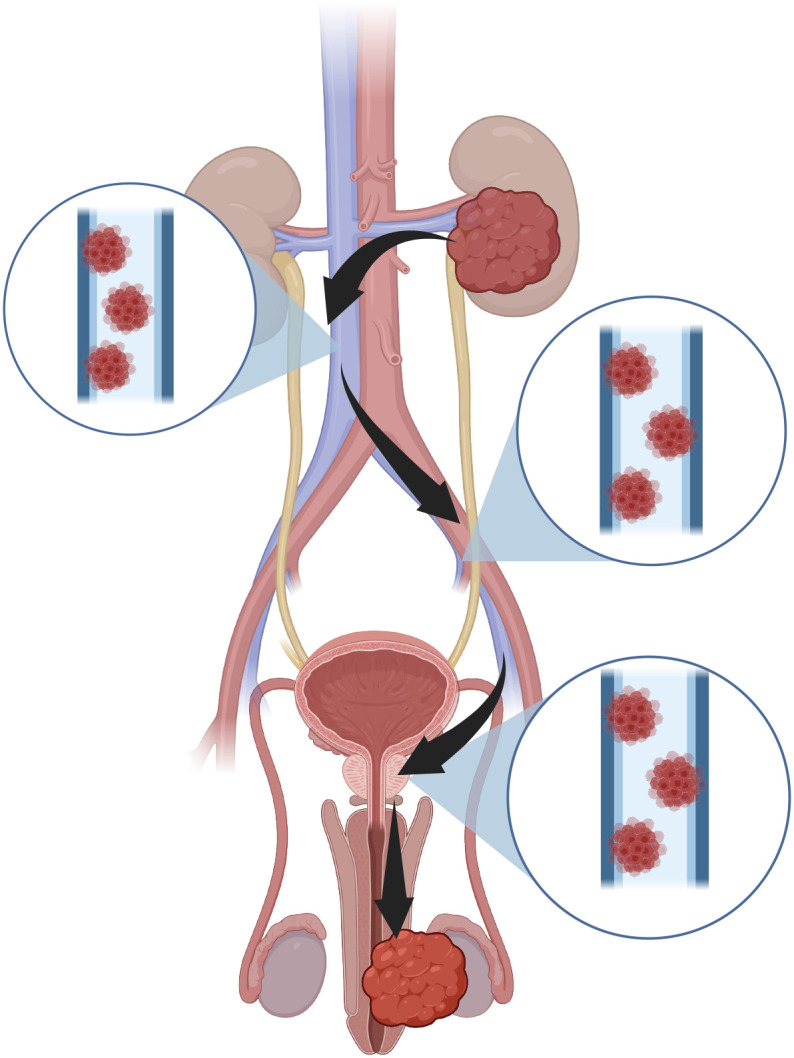
Schematic illustration of a hypothetical retrograde venous dissemination pathway from renal pelvic carcinoma to the penis.

Penile metastasis is a rare manifestation of disseminated malignancy (7–11). Reviews indicate that approximately 69% of penile metastases arise from genitourinary primaries, predominantly prostate (31%) and bladder (25%) cancers, followed by colorectal (13%) and renal (7%) origins (9–11). Renal pelvic SCC as a primary source of penile metastasis has not been previously documented. The main mechanism is retrograde venous dissemination through the pelvic venous plexus linking the dorsal penile vein to the prostatic and vesical venous systems (10, 12). Other possible routes include retrograde lymphatic spread, arterial dissemination, direct extension, and iatrogenic seeding (10, 13). In this case, the absence of pelvic or inguinal lymphadenopathy and the presence of hilar lymph node involvement suggest venous reflux as a plausible mechanism. Accordingly, we present a hypothetical venous route whereby tumor emboli travel from the renal vein → inferior vena cava → internal iliac → prostatic → dorsal penile venous plexus (**Figure 4**).

The strength of this report lies in the detailed pathological concordance between the renal pelvic and penile lesions and the imaging-based inference of a plausible metastatic route. However, this case has limitations, including the inability to completely exclude a synchronous primary penile carcinoma.

While p40/p63 expression reflects basal-type squamous differentiation, these markers are not site-specific and do not independently indicate metastatic potential. Therefore, their expression should be interpreted in conjunction with clinicopathological findings (4, 14). Loss of p16 expression distinguishes HPV-independent SCCs and supports chronic inflammation as the oncogenic driver (15). These molecular traits may explain the tumor’s unusual capacity for hematogenous spread to the penis.

## Conclusion

We describe, to our knowledge, the first documented case of penile involvement most consistent with metastatic renal pelvic SCC. The case highlights a rare but plausible route of dissemination via retrograde venous spread. Understanding these mechanisms is essential for appropriate diagnostic consideration and palliative management of penile lesions in patients with advanced upper urinary tract SCC.

## Data Availability

The original contributions presented in the study are included in the article/supplementary material. Further inquiries can be directed to the corresponding author.
